# Well Logging Based Lithology Identification Model Establishment Under Data Drift: A Transfer Learning Method

**DOI:** 10.3390/s20133643

**Published:** 2020-06-29

**Authors:** Haining Liu, Yuping Wu, Yingchang Cao, Wenjun Lv, Hongwei Han, Zerui Li, Ji Chang

**Affiliations:** 1 School of Geosciences, China University of Petroleum, Qingdao 266580, China; liuhaining632.slyt@sinopec.com (H.L.); caoych@upc.edu.cn (Y.C.); 2 Shengli Geophysical Research Institute of SINOPEC, Dongying 257022, China; hanhongwei.slyt@sinopec.com; 3 School of Electrical Engineering, Yanshan University, Qinhuangdao 066004, China; ypwu@zjut.edu.cn; 4 Department of Automation, University of Science and Technology of China, Hefei 230027, China; lzerui@mail.ustc.edu.cn (Z.L.); cjchange@mail.ustc.edu.cn (J.C.)

**Keywords:** lithology identification, domain adaptation, manifold regularization, projected maximum mean discrepancy, extreme learning machine

## Abstract

Recent years have witnessed the development of the applications of machine learning technologies to well logging-based lithology identification. Most of the existing work assumes that the well loggings gathered from different wells share the same probability distribution; however, the variations in sedimentary environment and well-logging technique might cause the data drift problem; i.e., data of different wells have different probability distributions. Therefore, the model trained on old wells does not perform well in predicting the lithologies in newly-coming wells, which motivates us to propose a transfer learning method named the data drift joint adaptation extreme learning machine (DDJA-ELM) to increase the accuracy of the old model applying to new wells. In such a method, three key points, i.e., the project mean maximum mean discrepancy, joint distribution domain adaptation, and manifold regularization, are incorporated into extreme learning machine. As found experimentally in multiple wells in Jiyang Depression, Bohai Bay Basin, DDJA-ELM could significantly increase the accuracy of an old model when identifying the lithologies in new wells.

## 1. Introduction

Well logging data have grown dramatically over the past few decades due to the widespread deployment of oil wells and the rapid development of sensing technology. A large amount of data not only brings us more opportunities to understand the underground but also brings more great challenges to the interpretation of logging data [1,2]. Well logging provides an objective and continuous method with which to observe the properties of the rocks through which the drill bit passes and to describe the deposition process quantitatively. As a bridge between surface geophysical survey and subsurface geology, well logging is an effective and irreplaceable method to understand reservoir characteristics. As conventional reservoirs dry up, oil and gas companies are turning to unconventional exploration and development in shale and low-permeability reservoirs, posing more challenges for logging interpretation [3].

In traditional logging interpretation, lithology determination, porosity, and permeability calculations are performed by specialists in exploration and geology with specialized knowledge. Lithology identification is a fundamental problem in well logging interpretation and is of considerable significance in petroleum exploration engineering. It is the basis of reservoir parameter calculation (such as porosity, shale volume, permeability) and geological research (such as formation correlation, sedimentary modeling, favorable zone prediction, etc.). Due to the complexity of reservoir geological conditions, the uncertainty of exploration data and the inconsistency of expert experience, the results of lithology identification are mainly dependent on expertise. With the increasing diversity of logging data, the traditional logging interpretation methods which rely on human experience have some shortcomings and limitations. As a result, researchers are turning to more advanced data analysis methods for breakthroughs in lithology identification.

In recent years, with the rapid development of machine learning technology, its application in lithology identification has also attracted full attention [4]. In order to accurately and effectively determine the lithology type, a large amount of application work based on different machine learning began to emerge [5]. For example, G. Askari [6] used satellite remote sensing data to find the lithological information in Deh-Molla sedimentary succession by principal component analysis. Al-Anazi et al. [7] proposed a support vector machine-based classification feature method and selection method based on fuzzy theory to realize the recognition of potential features and the improvement of lithology recognition performance. Wang et al. [8] proposed a novel back propagation (BP) model by modifying the self-adapting algorithm and activation function, which is proven to be effective in predicting the lithologies of the Kela-2 gas field. The experimental results show that, compared with discriminant analysis and probabilistic neural network, support vector machines can identify different lithology of heterogeneous sandstone reservoirs more accurately. Xie et al. [9] evaluated five typical machine learning methods of naive Bayes, support vector machines, artificial neural networks, random forest and gradient tree enhancement based on the formation lithology identification data of the Danudui gas field and the Hangjinqi gas field. Experimental results show that the integrated method, including random forest and gradient tree enhancement, has a lower prediction error. At the same time, the gradient tree enhancement method has the highest accuracy compared with the other four methods. In order to reduce exploration uncertainty, Bhattacharya et al. [10] compared different methods of facies division and prediction of mudstone in the current conventional logging data, and applied them to the Devonian Bakken and Mamentago–Marsilus formations in North America. In this study, support vector machines (SVM), artificial neural networks (ANN), self-organizing mapping (SFM), and multi-resolution map based clustering (MRMC) are compared experimentally. Dev et al. [11] analyzed the data from China’s Danuci gas field and Hangjinqi gas field by the gradient lifting decision tree system (i.e., XGBoost, LightGBM, and CatBoost) to study formation lithology classification, and compared their performance with random forest, AdaBoost, and gradient boosting machines. Experiments show that LightGBM and CatBoost are the preferred algorithms for lithology classification by using well logging data. A large number of similar studies can be seen in [12,13,14,15,16].

In addition to these directly-applied studies, more and more scholars are focusing on how to improve existing machine learning tools to solve practical problems in lithology identification. Due to the different distribution of underground lithology, there is a severe class imbalance problem in the training data set. Furthermore, Deng et al. [17] introduced a borderline-stroke technique for dealing with unbalanced data, and the results showed that this method could effectively improve the classification accuracy of SVM, especially for the minority classes. There are many free hyper-parameters in machine learning algorithms, and the settings of these hyper-parameters will have significant influences on the performance of lithology identification. Therefore, Saporetti et al. [18] adopted an evolutionary parameter tuning strategy, and combined gradient boosting (GB) with differential evolution (DE) to achieve the optimization of super parameters, thereby making the lithology identification more stable. In the study of [19], the wavelet decomposition was used to construct multi-channel images of logging data, and then the lithology identification problem based on the logging curve was skillfully transformed into an image segmentation problem. Finally, the feasibility of this method was verified by the application in the Daqing oilfield. Aiming at the issue of data drift between different wells, Ao et al. [20] proposed a hybrid algorithm for lithology identification that combines the mean drift algorithm and the random forest algorithm in the prototype similarity space. It is pointed out that a more accurate lithology identification model can be obtained by transforming the classification problem into prototype similarity space. Li et al. [21] proposed a semi-supervised algorithm based on Generated Adversarial Network, which uses logging curves as the labeled data and seismic data as the unlabeled data. The model was trained by Adam algorithm and uses the discriminator to identify the lithologies. Compared with the experimental results of various supervised methods, the model can effectively use unlabeled data to achieve higher prediction accuracy. Similar work can be found in [22,23,24].

**Figure 1:**
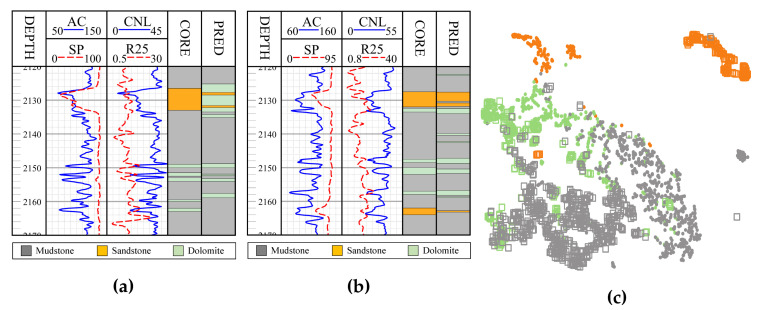
Illustration of data drift (**a**): Logging curves, core, and prediction results (using the model trained on well $W_{b}$) on well $W_{a}$. (**b**) Logging curves, core, and prediction results (using the model trained on $W_{a}$) of $W_{b}$. (**c**) t-SNE [25] visualization of data distribution for $W_{a}$ and $W_{b}$. Gray, yellow, and green indicate mudstone, sandstone, and dolomite, respectively. Point and hollow square indicate data from $W_{a}$ and $W_{b}$, respectively. $W_{a}$ and $W_{b}$ are geospatially near.

Although much work has been done, a practical problem, i.e., the well loggings from different wells are different in the probability distribution, is not taken into consideration. Hence, the model trained on old wells might not perform well on new wells. As illustrated in Figure 1, the phenomenon of data drift occurs between two wells, even when they are geospatially near. In particular, applying the model trained on well $W_{b}$ to well $W_{a}$ has many errors, as shown in Figure 1a. To suppress the data drift-induced accuracy decrease, we propose a transfer learning method named the data drift joint adaptation extreme learning machine (DDJA-ELM) to increase the accuracy of the old model applying to new wells. By incorporating the project mean maximum mean discrepancy, joint distribution domain adaptation, and manifold regularization into the extreme learning machine, we realize the knowledge transfer from old wells to new wells. As experimented in multiple wells in Jiyang Depression, Bohai Bay Basin, DDJA-ELM could increase the accuracy of an old model significantly when identifying the lithologies in new wells. In the remainder of the paper, Section 2 expatiates on the proposed DDJA-ELM, which is evaluated in Section 3. The last section concludes the paper.

## 2. Methodology

### 2.1. Notation

The dataset $D={\left\{\left(x_{i},y_{i}\right)\right\}}_{i=1}^{n}$ is composed of the sample $x_{i}={\left[x_{1},\ldots ,x_{d}\right]}^{\prime }\in R^{d}$ with *d* dimension and the label $y_{i}=\left[{\underset{︸}{0,\ldots ,0,1}}_{k},0,\ldots ,0\right]\in \left\{0,1\right\}$ if $x_{i}$ belongs to the *k*-th (1≤k≤c) class, where *n* and *c* are the numbers of samples and classes, respectively. In well logging-based lithology identification, *d* generally means the number of loggings types, and *c* denotes the number of lithology types. A sample is composed of the logging values at a certain depth. Considering the problem of data drift, we use $D_{S}$ and $D_{T}$ to differentiate the datasets with drift; i.e., $D_{S}$ and $D_{T}$ represent the source dataset for training and the target dataset to predict, respectively. Specifically, the source dataset is labeled and the target dataset is unlabeled, thereby denoting $D_{S}={\left\{\left(x_{i}^{s},y_{i}^{s}\right)\right\}}_{i=1}^{n_{s}}$ and $D_{T}={\left\{x_{i}^{t}\right\}}_{i=1}^{n_{t}}$ with $n_{s}$ and $n_{t}$ samples accordingly, and $n_{s}+n_{t}=n_{s+t}$, where $x_{i}^{s}$ and $x_{i}^{t}$ are source and target-dataset samples, respectively; and $y_{i}^{s}$ is a source-dataset label of $x_{i}^{s}$. By defining $X_{S}=\left[x_{1}^{s};\ldots ;x_{n_{s}}^{s}\right]$, $Y_{S}=\left[y_{1}^{s};\ldots ;y_{n_{s}}^{s}\right]$, and $X_{T}=\left[x_{1}^{t};\ldots ;x_{n_{t}}^{t}\right]$, then the $D_{S}=\left\{\left(X_{S},Y_{S}\right)\right\}$ and $D_{T}=\left\{X_{T}\right\}$.

### 2.2. Problem Definition and Formulation

In general, a classifier f(x) trained on D classifies well on those samples that satisfied the independent identically distributed (i.i.d.) assumption. However, the classifier f(x) trained on $D_{S}$ might not perform well on $D_{T}$ because the data drift means that the i.i.d. assumption does not hold; i.e., the knowledge learned from $D_{S}$ cannot transfer to $D_{T}$. In this case, it is necessary to add some new constraint conditions to achieve expected performance while learning the classifier *f*. Exactly, the expected performance can be concluded as the following constraint conditions: (i) minimizing the structural risk to avoid overfitting; (ii) minimizing the data drift between the source dataset and the target dataset; (iii) minimizing the prediction inconsistency within the target dataset. If considering the ELM as the basic classifier *f*, f(x)=f(x;β) where β is the output weight matrix of ELM.

According to the structural risk minimization (SRM) and regularization techniques [26], the classifier *f* can be denoted as

(1) $$f=\underset{\beta }{\arg\min}\ell \left(f\left(x;\beta \right),y\right)+R\left(x;\beta \right),$$

where the first term represents the empirical loss on samples (i.e., describing the fitness of applying the model to predict the training data); the second term represents the regularization term (i.e., representing the formulation of constraint conditions). Thus, combined with the constraint conditions mentioned above, the objective function is formulated mathematically as follows

(2) $$f=\underset{\beta }{\arg\min}\ell \left(f\left(x;\beta \right),y\right)+\frac{1}{2}{∥\beta ∥}^{2}+\frac{\lambda }{2}\Omega (D_{S},D_{T};\beta )+\frac{\gamma }{2}M\left(D_{T};\beta \right),$$

where the empirical loss term ℓ(f(x;β),y) and the structural risk regularization term ${∥\beta ∥}^{2}$ compose the model accuracy and complexity of ELM. The term $\Omega (D_{S},D_{T};\beta )$ indicates the extent of data drift between $D_{S}$ and $D_{T}$. Additionally, we introduce the manifold regularization term $M(D_{T};\beta )$ to improve the prediction consistency within the target dataset. λ and γ are the regularization parameters accordingly.

In the remainder of this section, we will expatiate on each term of the objective function.

#### 2.2.1. ELM

Recent years have witnessed the development of a promising machine learning model; i.e., extreme learning machine (ELM). ELM is actually an artificial neural network with a single hidden layer, which was first proposed by Huang et al. [27] and found its application in many domains, such as robotic perception, hyperspectral image classification, lithology identification, and human activity recognition [28,29,30,31,32]. Compared with support vector machine and other artificial neural networks, ELM has significant superiority in generalization performance and training time. In addition, many variants for ELM have been investigated, including semi-supervised ELM, multi-kernel ELM, rough ELM, one-class ELM, etc. [33,34,35,36].

According to the objective function (2), we first describe the mathematical model of ELM as follows [37],

(3a) $$\begin{array}{cc} \; & \underset{\beta \in R^{z\times c}}{\arg\min}\;\;\;\;\ell \left(f\left(x;\beta \right),y\right)+\frac{1}{2}{∥\beta ∥}^{2}=\frac{C}{2}\sum_{i=1}^{n_{s+t}}{∥e_{i}∥}^{2}+\frac{1}{2}{∥\beta ∥}^{2} \end{array}$$

(3b) $$\begin{array}{cc} \; & \;\;\;s.t.\;\;\;\;\;\;j_{i}h\left(x_{i}\right)\beta =y_{i}-e_{i},\;\;\;\;\;i=1,\ldots ,n_{s+t}, \end{array}$$

where $e_{i}\in R^{c}$ represents the error vector with respect to the *i*-th training sample, and $\sum_{i=1}^{n_{s+t}}{∥e_{i}∥}^{2}$ is the sum of prediction errors. The tradeoff coefficient *C* is used to balance the contribution between the two terms. Since the target dataset $D_{T}$ is unlabeled, $j_{i}=1$ if $i\leq n_{s}$; otherwise, $j_{i}=0$; and $y_{i}$ equals a zero vector if $i>n_{s}$. The output of hidden layer is equal to $h\left(x_{i}\right)=\left[g\left(x_{i};w_{1},b_{1}\right),\ldots ,g\left(x_{i};w_{z},b_{z}\right)\right],$ where $w_{j}\in R^{z}$; *z* is the number of hidden neurons; $b_{j}\in R$ are the *j*-th random generated weight vector and bias constant; g(·) is a piecewise continuous nonlinear activation function, such as the sigmoid function $g\left(x_{i};w_{j},b_{j}\right)=1/\left\{1+\exp\left(-w_{j}^{\prime }\cdot x_{i}-b_{j}\right)\right\}$ or the Gaussian function $g\left(x_{i};w_{j},b_{j}\right)=\exp(-b_{j}∥x_{i}-w_{j}{∥}^{2})$. In this paper, the sigmoid function is used as the activation function.

#### 2.2.2. Weighted ELM

Additionally, since each lithology in dataset D usually contains the samples of different amounts, appropriate weights should be assigned to each error vectors for the samples imbalance issue, so (3) is rewritten as

(4a) $$\begin{array}{cc} \; & \underset{\beta \in R^{z\times c}}{\arg\min}\;\;\;\;\ell \left(f\left(x;\beta \right),y\right)+\frac{1}{2}{∥\beta ∥}^{2}=\frac{C}{2}\sum_{i=1}^{n_{s+t}}\omega_{i}{∥e_{i}∥}^{2}+\frac{1}{2}{∥\beta ∥}^{2} \end{array}$$

(4b) $$\begin{array}{cc} \; & \;\;\;s.t.\;\;\;\;\;\;j_{i}h\left(x_{i}\right)\beta =y_{i}-e_{i},\;\;\;\;\;i=1,\ldots ,n_{s+t}, \end{array}$$

where $\omega_{i}=\frac{\nu_{i}}{\sum_{i=1}^{n_{s+t}}\nu_{i}}$ denotes the weight, $\nu_{i}=\frac{1}{n_{i}^{\tau }}$ and τ is a constant, and $n_{i}$ is the number of samples belonging to $y_{i}$, thereby modifying the ELM to the weighted ELM (WELM).

Substituting the constraints (4b) into (4a) yields the equivalent unconstrained optimization problem and the matrix form:

(5) $$\underset{\beta \in R^{z\times c}}{\arg\min}\;\frac{C}{2}W{∥(Y-JH\beta )∥}^{2}+\frac{1}{2}{∥\beta ∥}^{2},$$

where $W=\mathrm{blockdiag}\left(W_{S},0_{n_{t}\times n_{t}}\right)\in R^{n_{s+t}\times n_{s+t}}$; $W_{S}\in R^{n_{s}\times n_{s}}$ is a diagonal matrix with each item $\omega_{i}$; and $Y=\left[Y_{S};0_{n_{t}\times c}\right]\in R^{n_{s+t}\times c}$, $H=\left[h\left(x_{1}\right);\ldots ;h\left(x_{n_{s+t}}\right)\right]\in R^{n_{s+t}\times z}$, $J=\mathrm{blockdiag}\left(I_{n_{s}\times n_{s}},0_{n_{t}\times n_{t}}\right)\in R^{n_{s+t}\times n_{s+t}}$, where 0 and I are the zero matrix and the identity matrix with appropriate dimension, respectively. By setting the gradient of (5) over β to zero, we have

(6) $$\beta -CH^{\prime }J^{\prime }W\left(Y-JH\beta \right)=0,$$

where ′ represents the transpose of matrix. There are two forms of an optimal solution to the $\beta^{*}$. When the number of the training data is larger or smaller than the number of hidden neurons, then H has more or fewer rows than columns, thereby resulting in an underdetermined or overdetermined least squares problem. The closed-form solution for (6) can be described as

(7) $$\beta^{*}=\left\{\begin{array}{c} H^{\prime }W{\left(HH^{\prime }W+\frac{1}{C}I_{n_{s+t}}\right)}^{-1}Y,\;\;\mathrm{if}\;n_{s+t}<z\\ {\left(H^{\prime }WH+\frac{1}{C}I_{z}\right)}^{-1}H^{\prime }WY,\;\;\mathrm{if}\;n_{s+t}\geq z \end{array}\right.,$$

where $I_{n_{s+t}}$ and $I_{z}$ are the identity matrix of $n_{s+t}$ and *z* dimensions.

#### 2.2.3. Data Drift Adaptation

In general, the data distribution properties can be used to statistically describe the correlation among the samples $(x_{i},y_{i})\in D$, thus the data drift-induced distribution discrepancy can be estimated by statistical criteria. In this paper, we utilize the *projected maximum mean discrepancy* (PMMD) criterion for distribution discrepancy measure [38]. Thus, the regularization term $\Omega (D_{S},D_{T};\beta )$ with PMMD is formulated as

(8) $$\Omega_{P}\left(D_{S},D_{T};\beta \right)={∥\frac{1}{n_{s}}\sum_{i=1}^{n_{s}}h\left(x_{i}^{s}\right)\beta -\frac{1}{n_{t}}\sum_{j=1}^{n_{t}}h\left(x_{j}^{t}\right)\beta ∥}^{2},$$

Further, (8) can be rewritten to a matrix form; that is,

(9) $$\Omega_{P}\left(D_{S},D_{T};\beta \right)=\mathrm{tr}\left(\beta^{\prime }H^{\prime }M_{P}H\beta \right),$$

where tr(·) denotes the trace of a matrix. The elements of matrix $M_{P}\in R^{n_{s+t}\times n_{s+t}}$ are calculated by

(10) $$\begin{array}{c} {\left(M_{P}\right)}_{pq}=\left\{\begin{array}{cc} \frac{1}{n_{s}^{2}}, & \mathrm{if}\;\;p,q\leq n_{s}\\ \frac{1}{n_{t}^{2}}, & \mathrm{if}\;\;p,q>n_{s}\\ -\frac{1}{n_{s}n_{t}}, & \mathrm{otherwise}. \end{array}\right. \end{array}$$

According to (8), the samples $x_{i}^{s}$ and $x_{j}^{t}$ are transformed from the feature space to the mapping space by h(·), where the distribution discrepancy between the source and target datasets can be reduced by adjusting β. Thus, the modified WELM named data drift adaptation WELM (DDA-WELM) classifier can be achieved by introducing the regularization term $\Omega_{P}\left(D_{S},D_{T};\beta \right)$ to adapt the data drift.

However, the overall accuracy is increased with sacrificing the accuracies of some classes, because DDA-WELM is a method of reducing the distribution discrepancy between two datasets as a whole. Consequently, we modify the PMMD to propose the joint PMMD (JPMMD) that aims to reduce the distribution discrepancy between classes of two datasets, respectively. The regularization term $\Omega (D_{S},D_{T};\beta )$ with JPMMD is formulated as

(11) $$\Omega_{J}\left(D_{S},D_{T};\beta \right)=\sum_{k=1}^{c}{∥\frac{1}{n_{s}^{\left(k\right)}}\sum_{y_{i}^{s}=k}h\left(x_{i}^{s}\right)\beta -\frac{1}{n_{t}^{\left(k\right)}}\sum_{{\tilde{y}}_{j}^{t}=k}h\left(x_{i}^{s}\right)\beta ∥}^{2},$$

where $y_{i}^{s}$ is the real label of $x_{i}^{s}$ and ${\tilde{y}}_{j}^{t}$ is the pseudo label of $x_{j}^{t}$ which is generated by the DDA-WELM classifier; $n_{s}^{\left(k\right)}$ and $n_{t}^{\left(k\right)}$ are the number of source-dataset samples which belong to the class *k* and the number of target-dataset samples whose pseudo labels are *k*, respectively.

Similarly, (11) can be rewritten to the matrix form; that is,

(12) $$\Omega_{J}\left(D_{S},D_{T};\beta \right)=\mathrm{tr}\left(\beta^{\prime }H^{\prime }M_{J}H\beta \right),$$

where the elements of matrix $M_{J}\in R^{n_{s+t}\times n_{s+t}}$ are computed by

(13) $${\left(M_{J}^{\left(k\right)}\right)}_{pq}=\left\{\begin{array}{cccc} \; & \frac{1}{{\left(n_{s}^{\left(k\right)}\right)}^{2}}, & \; & \mathrm{if}\;\;p,q\leq n_{s}\;\mathrm{and}\;y_{p}^{s},y_{q}^{s}=k\\ \; & -\frac{1}{n_{s}^{\left(k\right)}n_{t}^{\left(k\right)}}, & \; & \mathrm{if}\;\;p>n_{s},q\leq n_{s}\;\mathrm{and}\;{\tilde{y}}_{p-n_{s}}^{t},y_{q}^{s}=k\\ \; & -\frac{1}{n_{s}^{\left(k\right)}n_{t}^{\left(k\right)}}, & \; & \mathrm{if}\;\;p\leq n_{s},q>n_{s}\;\mathrm{and}\;y_{p}^{s},{\tilde{y}}_{q-n_{s}}^{t}=k\\ \; & \frac{1}{{\left(n_{t}^{\left(k\right)}\right)}^{2}}, & \; & \mathrm{if}\;\;p,q>n_{s}\;\mathrm{and}\;{\tilde{y}}_{p-n_{s}}^{t},{\tilde{y}}_{q-n_{s}}^{t}=k\\ \; & 0, & \; & \mathrm{otherwise}. \end{array}\right.$$

Thus, the data drift joint adaptation WELM (DDJA-WELM) classifier can be obtained by introducing the regularization term $\Omega_{J}\left(D_{S},D_{T};\beta \right)$ and the DDA-WELM classifier to improve the WELM.

#### 2.2.4. Manifold Regularization

Manifold regularization is widely used to improve the smoothness of predictions and suppress the classifier cutting through the high-density regions [39]. Specifically, we introduce the manifold regularization $M(D_{T};\beta )$ to assign smooth labels within the target dataset and make the classifier more adaptable to the target dataset.

The formulation of the manifold regularization $M(D_{T};\beta )$ is that

(14) $$M\left(D_{T};\beta \right)=\frac{1}{2n_{t}^{2}}\sum_{i=1}^{n_{t}}\sum_{j=1}^{n_{t}}a_{i,j}{∥h\left(x_{i}^{t}\right)\beta -h\left(x_{j}^{t}\right)\beta ∥}^{2}$$

where the similarity $a_{i,j}$ between the sample $x_{i}^{t}$ and $x_{j}^{t}$ is calculated by

(15) $$\begin{array}{c} a_{i,j}=\left\{\begin{array}{cccc} \; & \exp\left(\frac{-{∥x_{i}^{t}-x_{j}^{t}∥}^{2}}{4\sigma^{2}}\right), & \; & x_{i}^{t}\in N\left(x_{j}^{t}\right)\mathrm{or}x_{j}^{t}\in N\left(x_{i}^{t}\right),\\ \; & 0, & \; & \mathrm{otherwise}, \end{array}\right. \end{array}$$

where $N(x_{j}^{t})$ is the set of κ-nearest neighboring samples of $x_{i}^{t}$ under the metric of Euclidean distance in feature space, and σ>0 is the width of Guassian kernel. Additionally, (14) can be rewritten to a matrix form

(16) $$M\left(D_{T};\beta \right)=\mathrm{tr}\left(\beta^{\prime }H^{\prime }LH\beta \right).$$

where $L=\mathrm{diag}\left(0_{n_{s}\times n_{s}},L_{T}\right)\in R^{n_{s+t}\times n_{s+t}}$, $L_{T}=D-A$ is the target dataset Laplacian matrix, and $A={\left[a_{i,j}\right]}_{n_{t}\times n_{t}}$, D is a diagonal matrix with diagonal elements $d_{i}=\sum_{j=1}^{n_{t}}a_{i,j}$.

Thus, the classifiers DDA-WELM and DDJA-WELM can be upgraded to the DDA-S2WELM and DDJA-S2WELM by introducing the manifold regularization term $M(D_{T};\beta )$. Moreover, the regularization term $\Omega_{J}\left(D_{S},D_{T};\beta \right)$ with pseudo labels generated by the DDA-S2WELM classifier can further improve the data drift adaptation performance of the DDJA-WELM and DDJA-S2WELM classifiers.

### 2.3. Solution of Objective Function

The solution of the objective function (2) is introduced in this section. Especially, the regularization term $\Omega (D_{S},D_{T};\beta )$ is divided into $\Omega_{P}\left(D_{S},D_{T};\beta \right)$ with PMMD and $\Omega_{J}\left(D_{S},D_{T};\beta \right)$ with JPMMD so that we will discuss them in the followings, respectively.

#### 2.3.1. Solution of Objective Function with $\Omega_{P}$

By incorporating (5), (9), and (16) into (2), we have

(17) $$f=\underset{\beta \in R^{z\times c}}{\arg\min}\frac{C}{2}W{∥Y-JH\beta ∥}^{2}+\frac{1}{2}{∥\beta ∥}^{2}+\frac{\lambda }{2}\mathrm{tr}\left(\beta^{\prime }H^{\prime }M_{P}H\beta \right)+\frac{\gamma }{2}\mathrm{tr}\left(\beta^{\prime }H^{\prime }LH\beta \right).$$

By setting the gradient of *f* with respect to β to be zero

(18) $$\nabla f=CH^{\prime }J^{\prime }W\left(JH\beta -Y\right)+\beta +\lambda H^{\prime }M_{P}H\beta +\gamma H^{\prime }LH\beta =0.$$

According to (18), the closed-form solution of the optimal β is

(19) $$\beta^{*}=\left\{\begin{array}{c} H^{\prime }W{\left(\frac{1}{C}I_{n}+\left(W+\frac{\lambda }{C}M_{P}+\frac{\gamma }{C}L\right)HH^{\prime }\right)}^{-1}Y,\;\;\mathrm{if}\;n_{s}+n_{t}<z\\ {\left(\frac{1}{C}I_{z}+H^{\prime }\left(W+\frac{\lambda }{C}M_{P}+\frac{\gamma }{C}L\right)H\right)}^{-1}H^{\prime }WY,\;\;\mathrm{if}\;n_{s}+n_{t}\geq z \end{array}\right.$$

where *z* is the number of hidden neurons.

#### 2.3.2. Solution of Objective Function with $\Omega_{J}$

Similarly, incorporating (5), (12), and (16) into (2), and solving $\frac{\partial f}{\partial \beta }=0$ yields the optimal β as follows

(20) $$\beta^{*}=\left\{\begin{array}{c} H^{\prime }W{\left(\frac{1}{C}I_{n}+\left(W+\frac{\lambda }{C}M_{J}+\frac{\gamma }{C}L\right)HH^{\prime }\right)}^{-1}Y,\;\;\mathrm{if}\;n_{s}+n_{t}<z\\ {\left(\frac{1}{C}I_{z}+H^{\prime }\left(W+\frac{\lambda }{C}M_{J}+\frac{\gamma }{C}L\right)H\right)}^{-1}H^{\prime }WY,\;\;\mathrm{if}\;n_{s}+n_{t}\geq z \end{array}\right.$$

where *z* is the number of hidden neurons.

## 3. Experimental Verification

In this section, we conduct extensive experiments to verify the effectiveness of our method, using the well-logging data collected from multiple wells in Jiyang Depression, Bohai Bay Basin. The experimental datasets and settings will be described first. Then, the performance and impact of each regularization terms are shown in detail. The analysis of hyper-parameters sensibility is presented at last.

### 3.1. Experimental Settings

**Figure 2:**
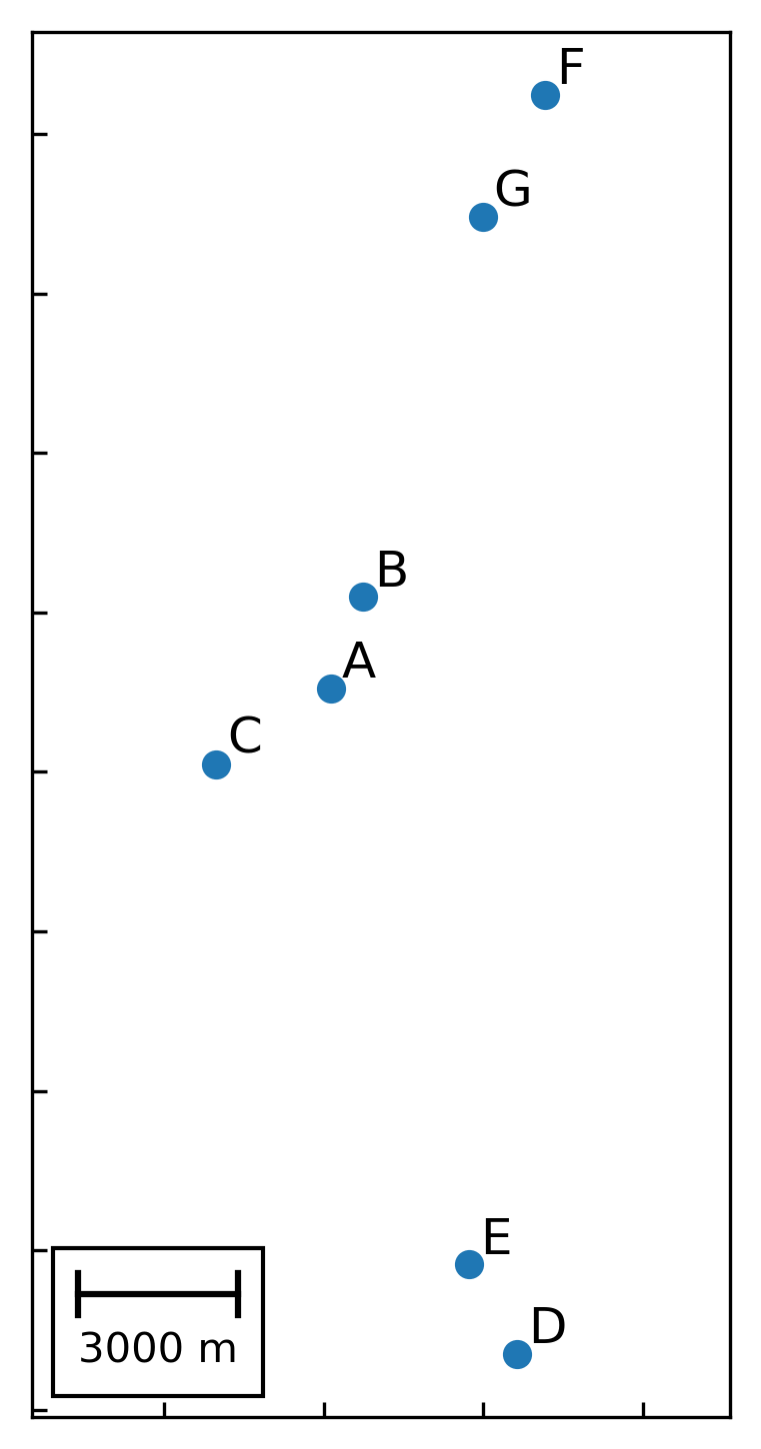
Relative positions of experimental wells.

As shown in Table 1, the experimental datasets are composed of three datasets collected from different regions in the Jiyang Depression, Bohai Bay Basin and contain 3, 2 and 2 wells, respectively (The wells in Figure 1 do not appear in the experimental datasets). Their relative positions are shown in Figure 2. Table 2 describes the data drift statistically by maximum mean discrepancy. In one experiment, we set the well A as the training data and the well B as the testing data, thereby verifying the effectiveness of our method through A → B. In this case, the other experiments are denoted as A → C, B → A, B → C, C → A, C → B, D → E, E → D, F → G, and G → F, respectively. Considering the different ranges of measurements, the value of samples are transformed to [0, 1] by a min-max normalization method. In the experiments, we adopt the index Recall (i.e., the number of classify correctly divided by the number of samples) to calculate the classification accuracy, thus denoting the average recall (Macro-R) to represent the overall classification accuracy.

### 3.2. Experimental Results

**Figure 3:**
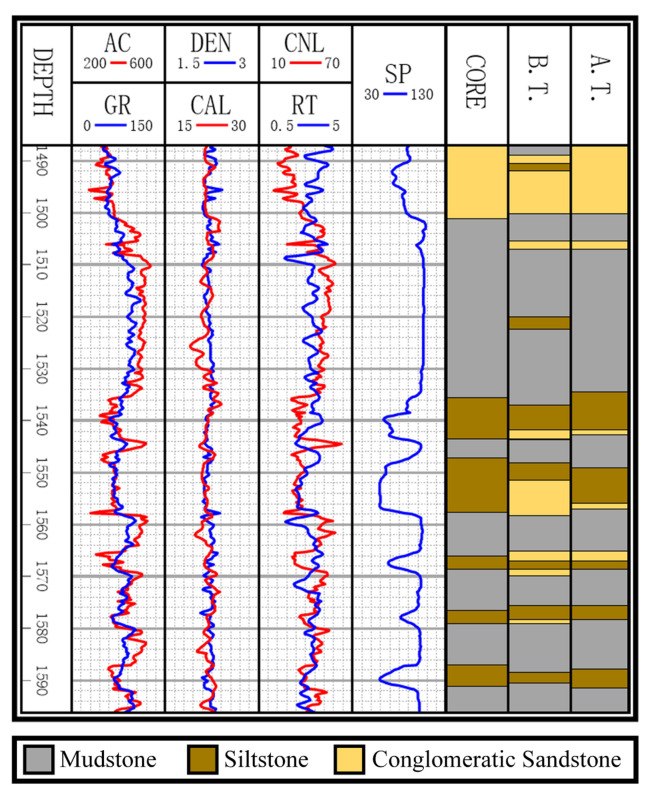
Logging curves, core, and classification results of B.T. (before transfer) and A.T. (after transfer).

**Figure 4:**
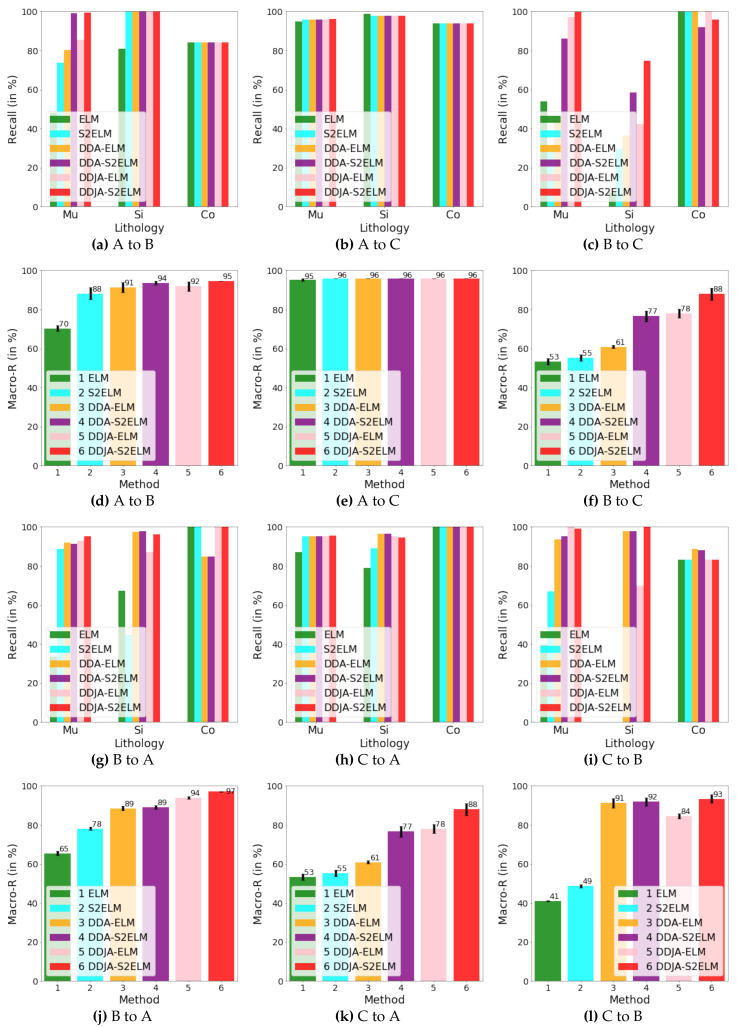
The results of performing our method on dataset 1. In (**a**)–(**c**), (**g**)–(**i**), we show the recalls for the classes of Mu, Si, and Co by using different methods. In (**d**)–(**f**), (**j**)–(**l**), we show the macro average recalls by using different methods.

**Figure 5:**
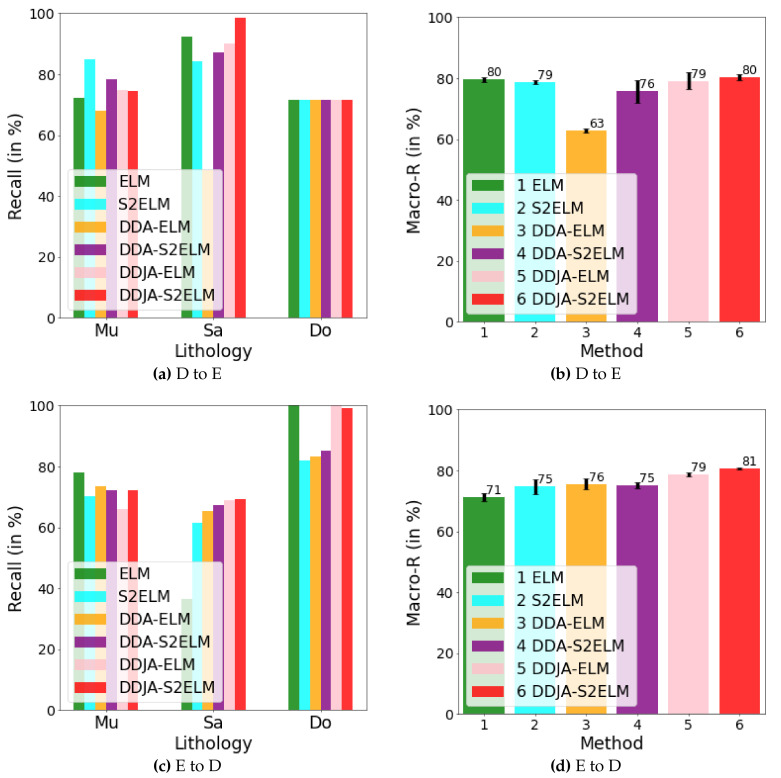
The results of performing our method on dataset 2. In (**a**) and (**b**), we show the recalls for the classes of Mu, Sa, and Do and their macro average recall by transferring D to E. In (**c**) and (**d**), we show those by transferring E to D.

**Figure 6:**
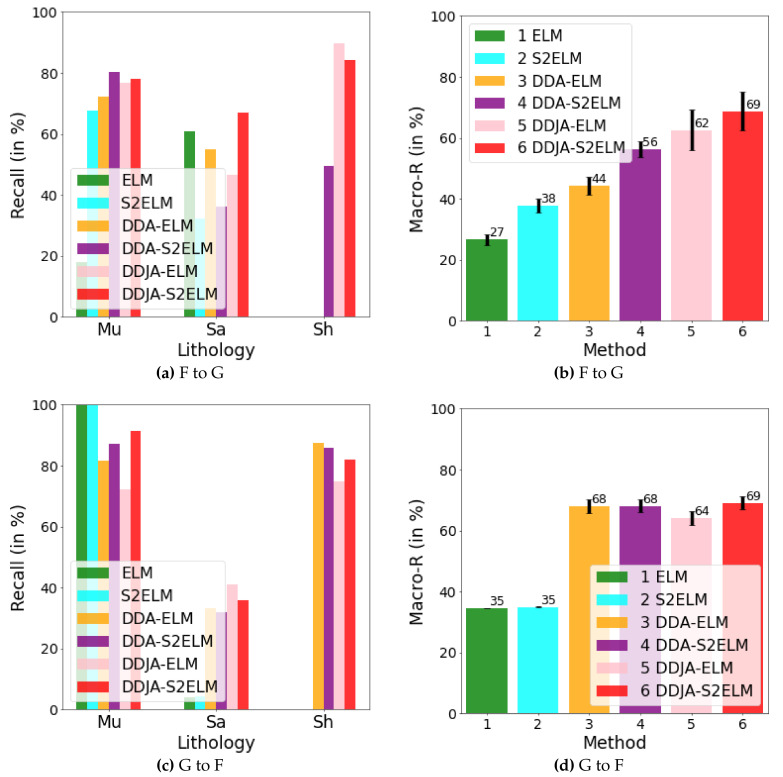
The results of performing our method on dataset 3. In (**a**) and (**b**), we show the recalls for the classes of Mu, Sa, and Sh and their macro average recall by transferring F to G. In (**c**) and (**d**), we show those by transferring G to F.

Figure 3 exhibits the logging curves, core (i.e., label), classification performance before B → A transfer (i.e., using model trained on data B to directly predict data A), and classification performance after B → A transfer. It is observed that transfer learning could eliminate the data drift-induced accuracy loss. As shown in Figure 4, Figure 5, and Figure 6, the classification performances of applying the classifiers ELM, S2ELM, DDA-ELM, DDA-S2ELM, DDJA-ELM, and DDJA-S2ELM to datasets 1, 2, and 3 are presented, respectively. It can be obviously observed from Figure 4a–c,g–i, Figure 5a,c, and Figure 6a,c, that the accuracy of each class is gradually increased with the introducing of DDJA regularization terms, especially when it comes to the accuracy of Si and Sh that increase from 0 to more than 80 in Figure 4i, Figure 6a,c, respectively. Additionally, the ELM and S2ELM classifiers without DDJA regularization term are insufficient to the tasks with data drift.

Considering the ELM–based classifier with the random weights and biases parameters, we conduct multiple experiments by setting different random seeds to generate different weights and biases parameters which yield the experimental results in Figure 4d–f,j–l, Figure 5b,d and Figure 6b,d. According to the results, we have the following observations: (i) The overall Macro-R shows a trend of increasing step by step and the DDJA-S2ELM classifiers achieve the highest accuracy. Moreover, the Macro-R of DDJA-S2ELM are increased to 88% at least compared with the ELM on dataset 1 (B → C and C→ A), 80% on dataset 2 (D → E), and 69% on dataset 3 (F →G and G → F). Additionally, compared the ELM classifier with the DDJA-S2ELM classifier, the Macro-R of our method are increased 52% on dataset 1 (C→ B), 10% on dataset 2 (E→ D), and 42% on dataset 3 (F→ G). (ii) Although the Macro-R is not increased on dataset 1 (A → C) and dataset 2 (D → E), the standard deviation can be kept at a lower level. Thus, the stability can be improved by introducing the DDJA-S2ELM. (iii) Comparing the experimental results on dataset 3 with datasets 1 and 2, the ELM classifiers only achieve 27% (F→ G) and 35% (G→ F). The performance is significantly enhanced with our method regarding the data drift-induced accuracy decrease surges.

### 3.3. Parameters’ Sensitivity

In this section, we aim to analyze the sensitivity on the key hyper-parameters of the DDJA-S2ELM: the trade-off coefficient *C*, the contribution coefficient of DDJA regularization term λ, and the contribution coefficient of semi-supervised regularization term γ. By analyzing the Macro-R over different settings of hyper-parameters, the configuration of these parameters is given. To avoid repetition, we only show the results of B → C.

**Figure 7:**
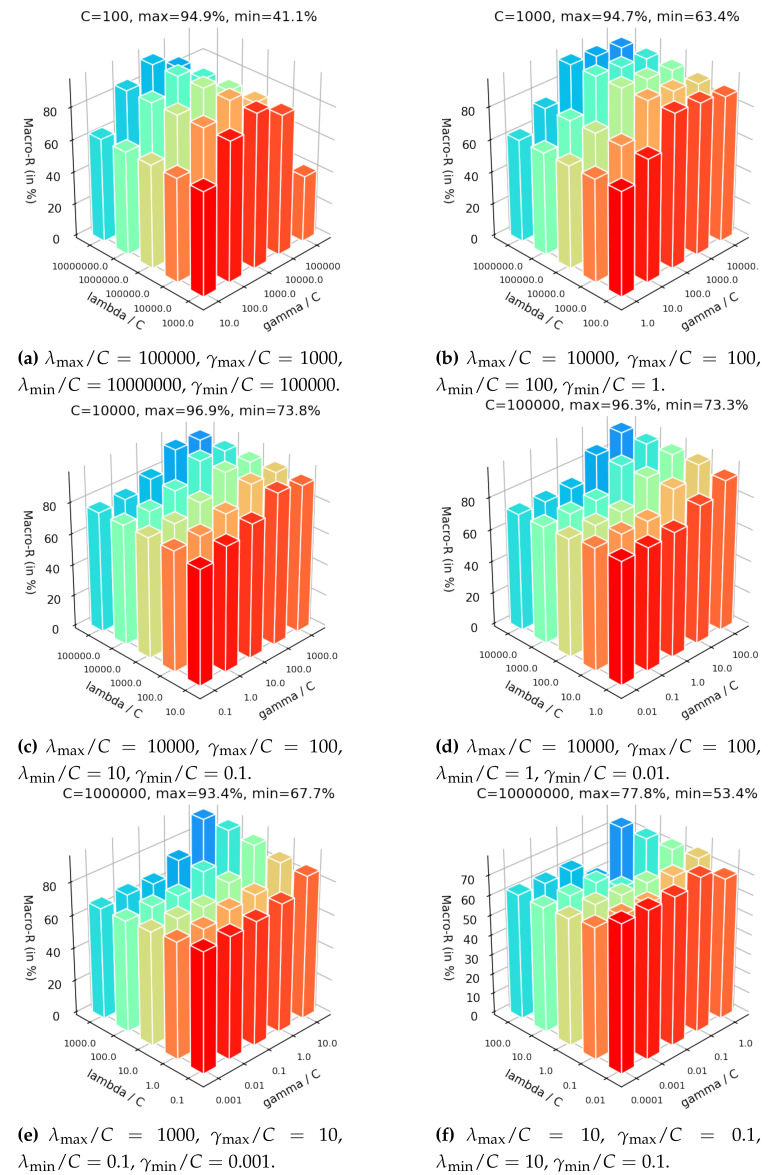
Classification accuracies of JDA-S2ELM on dataset 1 over the variation of *C*, λ and γ.

In the Figure 7, we set the hyper-parameter *C* range from 100 to 10,000,000. It can be seen from these figures in Figure 7 that the maximum and minimum Macro-R are increased first and then decreased with the increasing of *C*, indicating that a small *C* incurs under-fitting in classification and a high *C* causes the over-fitting. Additionally, when *C* is set too small or too big, the overall accuracies are preserved in the large variation range highest 94.9% in Figure 7a and lowest 41.1% in Figure 7a or the overall just more than 70% sightly in Figure 7f. According to Figure 7c,d with C=1000 and C=10000, respectively, the overall Macro-R are held steady highest 96.9% in Figure 7c and lowest 73.8% in Figure 7c and the accuracies are presented a trend of increasing gradually. Therefore, it is important to configure a moderate *C* first.

The influence of adjusting λ and γ are given by fixing the *C*. It can be seen from Figure 7 that the maximum Macro-R is achieved 96.9% in Figure 7c when set λ/C=10,000 and γ/C=100. Additionally, the Macro-R almost increase first and then decrease with γ getting larger under fixed λ showing in Figure 7a–c. Moreover, these results show that the maximum is achieved almost when setting the λ larger than γ by two to four orders of magnitude. Since the γ control the contribution of semi-supervised regularization term that based on the manifold assumption, but the assumption is invalid when the dataset with data drift. We introduce the DDJA regularization term to suppress the data drift, so the coefficient λ should be more than γ.

According to the analysis of setting *C*, λ, and γ mentioned above, it can be concluded that: (i) *C* should be set a relatively larger range first to configure a moderate value; (ii) the setting of λ should be bigger than γ by two to four orders of magnitude. Here are some suggested settings: $C\in [10^{3},10^{6}]$, $\frac{\lambda }{C}\in \left[10^{3},10^{4}\right]$, $\frac{\gamma }{C}\in \left[10^{2},10^{3}\right]$.

Furthermore, we use Sobol method (In our experiment, the python tool "SALib" is employed, which can be found at https://salib.readthedocs.io/en/latest/index.html). Ref. [41] to implement a global sensitivity analysis where all parameters are varied simultaneously over the entire parameter space. The sensitivities shown in Table 3 demonstrate that: (i) *C* and λ contribute mainly compared with γ, (ii) *C* contributes more than λ slightly, and (iii) the interactions between these parameters are weak, so they are relatively independent.

## 4. Conclusions

In this paper, we have investigated the well logging-based lithology identification under data drift, thus proposing a transfer Extreme Learning Machine method to handle it. According to the projected maximum mean discrepancy (PMMD) criterion and extreme learning machine, we introduce a new PMMD criterion and then propose the DDJA-ELM to minimize the data drift between the source dataset and the target dataset. Additionally, in order to improve the prediction consistency within the target dataset, the manifold regularization is introduced to promote the DDJA-ELM to the DDJA-S2ELM. Extensive experiments have validated the high and stable accuracy of our method.

## Figures and Tables

**Table 1:** Dataset description.

Dataset	1				2				3			
LOGS	AC, CAL, CNL, GR, RT, SP				AC, CAL, GR, R25, SP				AC, CAL, GR, R25, SP			
Samples	LITH	Mu	Si	Co	LITH	Mu	Sa	Do	LITH	Mu	Sa	Sh
	Well A	3820	2048	452	Well D	4508	3076	1000	Well F	6072	2852	1644
	Well B	2164	1584	432	Well E	5000	1464	1112	Well G	6996	3404	1548
	Well C	2368	1452	400	-				-			

[1] Logging curves. AC: acoustic log, CAL: caliper log, CNL: compensated neutron log, GR: gamma ray log, RT: true formation resistivity, SP: spontaneous potential log, R25: 2.5 m bottom gradient resistivity. [2] Lithology. Mu: mudstone, Si: siltstone, Co: conglomeratic sandstone, Sa: sandstone, Do: dolomite, Sh: shale.

**Table 2:** Data drift description.

	A and B	A and C	B and C	D and E	F and G
MMD Class 1	0.107	0.182	0.281	0.109	0.090
MMD Class 2	0.230	0.067	0.277	0.238	0.251
MMD Class 3	0.203	0.026	0.384	0.189	0.601
MMD Overall	0.540	0.275	0.942	0.536	0.942

[1] MMD is short for maximum mean discrepancy, which can be found in [40]. [2] For (A,B), (A,C), and (B,C), classes 1–3 mean Mu, Si, and Co, respectively. For (D,E), classes 1–3 mean Mu, Sa, and Do, respectively. For (F,G), classes 1–3 mean Mu, Sa, and Sh, respectively.

**Table 3:** Sensitivity analysis using Sobol method.

Param.	First-Order Sensitivity	Total Sensitivity		Param. 1	Param. 2	Second-Order Sensitivity
*C*	0.511	0.575		*C*	λ	0.074
λ	0.337	0.495		*C*	γ	0.008
γ	0.051	0.102		λ	γ	0.085
